# Impact of Various Moisture Control Methods on Intraoral Humidity

**DOI:** 10.3290/j.jad.c_2398

**Published:** 2025-12-11

**Authors:** Takashi Washino, Masaomi Ikeda, Michael F. Burrow, Toru Nikaido

**Affiliations:** a Takashi Washino Visiting Lecturer, Department of Operative Dentistry, Division of Oral Functional Science and Rehabilitation, School of Dentistry, Asahi University, 1851 Hozumi, Mizuho 501-0296, Japan. Conducted the research and wrote the manuscript.; b Masaomi Ikeda Professor, Department of Oral Biomedical Engineering, Graduate School of Medical and Dental Sciences, Institute of Science Tokyo, 1-5-45 Yushima, Bunkyo-ku, Tokyo 113-8549, Japan. Performed the statistical analysis.; c Michael F. Burrow Clinical Professor, Associate Dean, Restorative Dental Sciences, Faculty of Dentistry, The University of Hong Kong, Prince Philip Dental Hospital, 34 Hospital Road, Sai Ying Pun, Hong Kong SAR, China. Reviewed and checked the manuscript.; d Toru Nikaido Professor, Asahi University, 1851 Hozumi, Mizuho 501-0296, Japan. Supervised and oversaw the entire project.

**Keywords:** adhesive dentistry, intraoral humidity, moisture control, rubber dam, vacuum-assisted isolation

## Abstract

**Purpose:**

This prospective crossover clinical study evaluated the effectiveness of different moisture control methods on intraoral humidity and examined the influence of external environmental humidity.

**Materials and Methods:**

Forty adult participants (31 females, 9 males; mean age 36.6 ± 11.3 years) were enrolled. Intraoral relative humidity was measured above anterior and posterior teeth under four randomized conditions: control (mouth breathing), rubber dam isolation, vacuum-assisted isolation (ZOO), and saliva ejector. External humidity was recorded simultaneously and categorized as high (≥60%) or low (<60%).

**Results:**

Both rubber dam isolation and vacuum-assisted isolation significantly reduced intraoral humidity compared with the saliva ejector and control. Under low external humidity, rubber dam and vacuum-assisted isolation achieved the lowest intraoral humidity (all P <0.001). Under high external humidity, all methods reduced humidity compared with control, but vacuum-assisted isolation remained significantly more effective than the rubber dam and saliva ejector. External humidity showed a strong correlation with intraoral humidity in the rubber dam group (ρ up to 0.77, P <0.001).

**Conclusion:**

Moisture control methods differ in their ability to reduce intraoral humidity, with rubber dam and vacuum-assisted isolation providing the most effective reduction. However, high external humidity significantly increases intraoral humidity regardless of the isolation method, indicating that clinicians should consider both the isolation strategy and environmental conditions when aiming to minimize intraoral humidity.

Intraoral humidity varies depending on exhalation and inhalation; during exhalation, a substantial increase in humidity occurs.^17^ Direct composite restorations and indirect metal-free restorations rely heavily on secure bonding for long-term clinical success, and high humidity can compromise bonding strength.

Previous studies have shown that high-humidity environments adversely affect bonding performance. Falacho et al^6^ demonstrated that rubber dam isolation improves enamel bond strength, whereas Burrow et al^4^ and Jacquot et al^11^ observed increased adhesive failures, reduced bond strength, and greater microleakage under high humidity. Washino et al^22^ reported that bonding performance differed depending on the type of resin cement in dentin bonding tests under high humidity, emphasizing the importance of moisture control. Humidity impairs adhesion by leaving residual water films that hinder resin infiltration and solvent/monomer evaporation, leading to phase separation, a lower degree of conversion, and increased failures.

Furthermore, systematic reviews and meta-analyses support the clinical relevance of humidity control. Wang et al^21^ and Miao et al^15^ concluded that rubber dam isolation may increase short-term survival of restorations, though the certainty of evidence was low. Mahn et al^13^ and Heintze et al^9^ highlighted that both adhesive type and rubber dam isolation significantly influence clinical outcomes. Despite this recognition, few studies have directly quantified intraoral humidity under different isolation methods in clinical settings.

Therefore, this study aimed to evaluate the effectiveness of various humidity control methods and the influence of external environmental factors in a clinical situation. The hypothesis was that conventional and advanced humidity control methods would reduce intraoral humidity under intra- and extraoral conditions.

## MATERIALS AND METHODS

### Participants

This was a prospective crossover clinical study conducted in a private dental clinic setting between June 24, 2022, and March 31, 2023. A total of 45 adult participants who visited the dental clinic were recruited. After screening based on predefined criteria, 40 participants were included in the final analysis. Inclusion criteria (adapted from Kameyama et al and inferred from the present exclusion list) were: adults in good general health or with noncontributory medical conditions, with sound dentition and occlusion, and without extensive prostheses.

The exclusion criteria were as follows^11^:

Missing posterior teeth or the use of extensive prostheses could affect humidity measurements.
History of temporomandibular disorders (TMD) or discomfort during prolonged mouth opening.
Reduced salivary flow rate (<0.5 ml/min) or xerostomia-related conditions.
Severe tooth sensitivity or discomfort during restorative procedures.
Nasal or throat disorders that could interfere with mouth-breathing control.
Latex or silicone allergies, particularly relevant for rubber dam application.
Pregnancy or other medical conditions requiring special precautions.

Informed consent was obtained from all participants, and approval was granted by the [blinded for review] Ethics Committee (Approval No. [blinded for review]). The final sample consisted of 40 participants (31 females and 9 males) with a mean age of 36.6 ± 11.3 years (range, 21–72 years).

### Moisture Control Methods

The following four conditions were assessed. All four conditions were performed in every participant for both an anterior (left maxillary central incisor) and a posterior tooth (left mandibular first molar). The order of the four conditions was randomized by drawing numbered chopsticks (lottery method).

**Fig 1:**
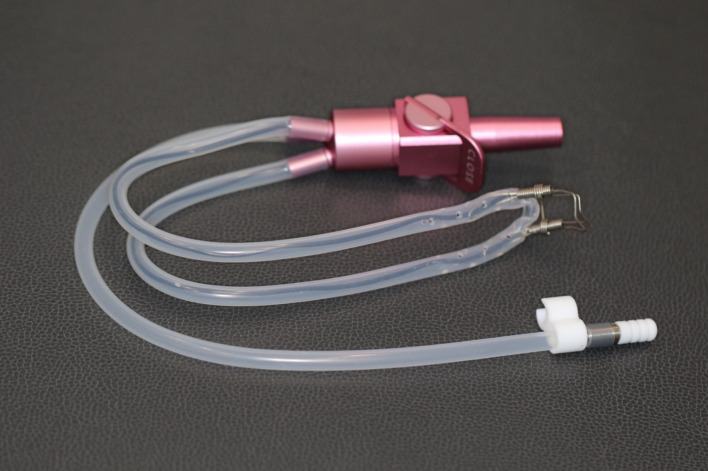
ZOOα mini.

1. Control group (C): Oral breathing with mouth open
  Participants kept their mouths open and breathed orally without any additional humidity/moisture control.
2. Rubber dam group (RD):
  For molars, an RD was applied using a Dentek #56 clamp (Dentek, Tokyo, Japan) to isolate a single tooth (left mandibular first molar).
  For anterior teeth, an RD was applied using a YDM #201 clamp (YDM, Tokyo, Japan) to isolate a single tooth (left maxillary central incisor).
  The RD and clamp were carefully secured using a standardized protocol, and the area around the tooth was sealed with a light-cured sealant (MEDICLUS, Cheongju, Korea) to prevent leakage and ensure proper isolation.
  A thin RD sheet (Blossom Dental, Taipei, Taiwan) was used.
3. Vacuum-assisted isolation group (ZOO):
  Figure 1 presents the ZOO α mini (APT Co, Osaka, Japan). The ZOO α mini device, a vacuum-assisted isolation system, was placed buccally and lingually around the left mandibular first molar or the left maxillary central incisor to surround the measurement site, and
  The device was connected to a vacuum unit integrated in the same dental chair (KaVo E50; KaVo Dental, Biberach, Germany) to continuously remove moisture from the oral cavity.
4. Saliva ejector group (SE):
  A standard bent saliva ejector (FEED Saliva Ejector; FEED, Tokyo, Japan) was used and placed at the right oral commissure,
  The saliva ejector was connected to an oral evacuation system and maintained at a constant flow to effectively remove saliva around the measurement area.

### Intraoral Humidity Measurement

**Fig 2:**
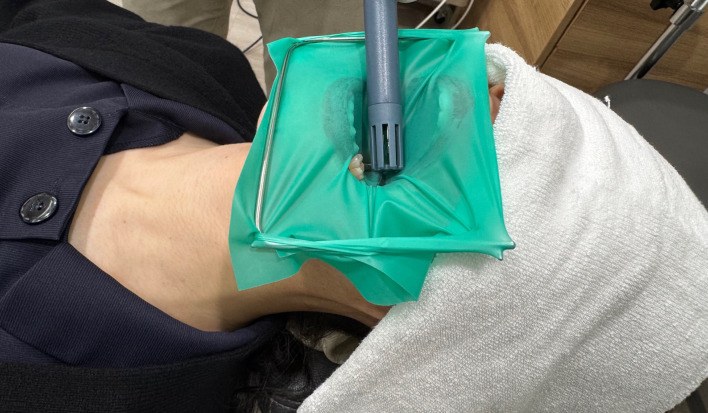
Measuring intraoral humidity using a hygrometer.

Figure 2 shows the experimental conditions. Measurements were conducted by a single experienced dentist using a digital hygrometer (Model CTH-1100, Custom Corp, Tokyo, Japan) positioned 1 cm above the tooth surfaces (left maxillary central incisor for anterior and left mandibular first molar for posterior). Readings were taken four times at 10-s intervals, and the average value was recorded. Participants were instructed to breathe through the mouth during all measurements, and nasal breathing was minimized through verbal guidance; no physical devices were used.

All participants were positioned in a supine position on the dental chair and were verbally instructed to maintain oral breathing during measurements. All measurements were performed by the same operator in the same operatory room to minimize variability, although measurements were not restricted to the same time of day.

### Extraoral Humidity Measurement

Environmental humidity was measured using the same digital humidity and temperature meter positioned 30 cm from the participant’s mouth. Humidity levels above 60% were classified as high (H), while those below 60% were classified as low (L). All measurements were conducted in the same operatory using a single dental unit to standardize the suction environment.

### Statistical Analysis

Post-hoc analyses were conducted using G*Power 3.1.9.7 to evaluate effect sizes and statistical power. Spearman’s rank correlation coefficient was used for correlation analysis. Wilcoxon signed-rank tests with Bonferroni correction were applied for intergroup comparisons. Statistical significance was set at P <0.05.

## RESULTS

The study population included 40 participants (31 females and 9 males) with a mean age of 36.6 ± 11.3 years (range, 21–72 years).

### Intraoral Humidity Under Different Conditions

**Fig 3a to h:**
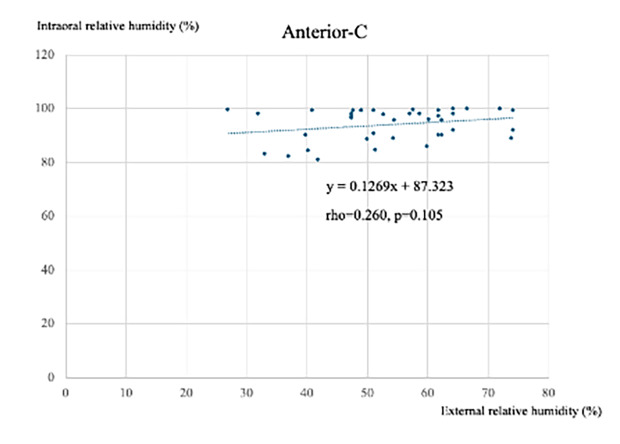

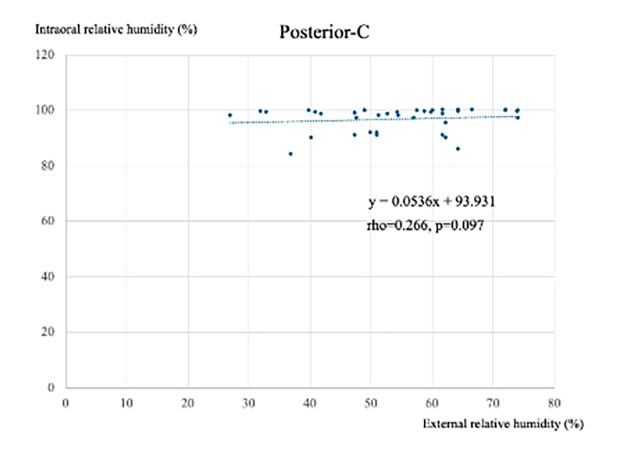

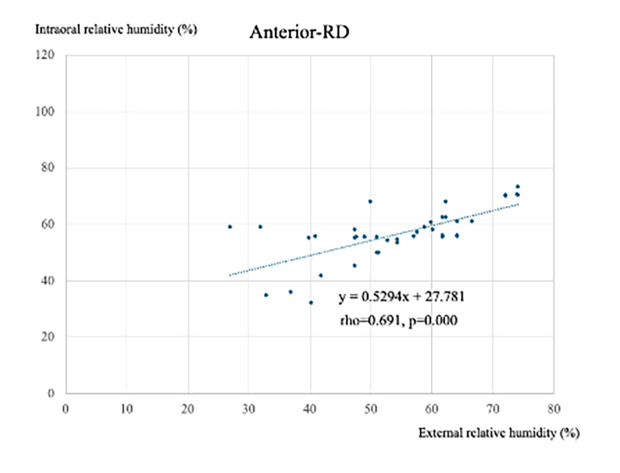

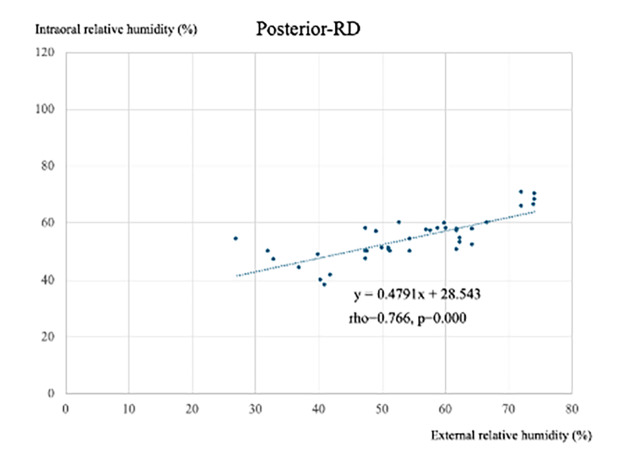

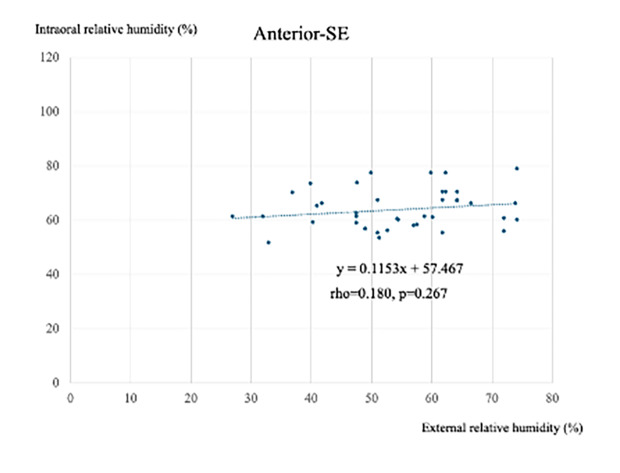

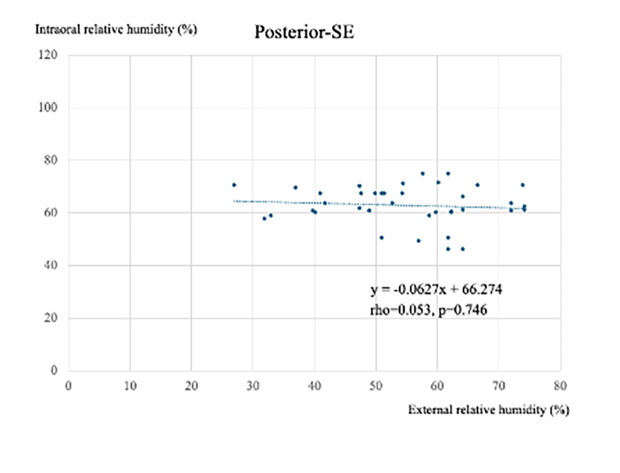

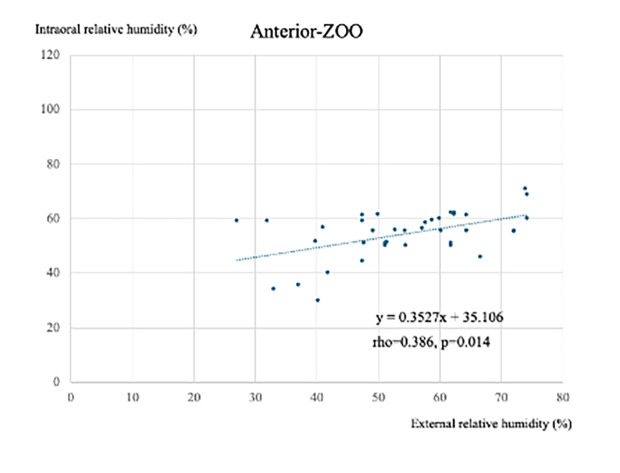

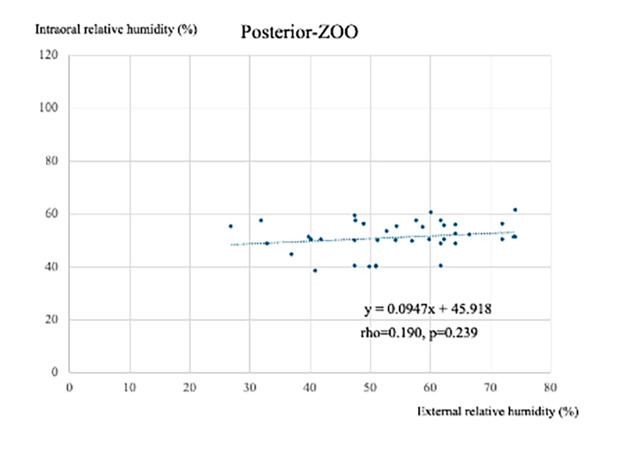
Intraoral humidity levels (unit: %) under different moisture control methods in anterior and posterior regions.

Figure 3 shows the intraoral humidity levels for each moisture control condition. No significant differences in intraoral humidity were found between anterior and posterior teeth for any moisture control method (P >0.05).

Under high external humidity (H), intraoral humidity levels for anterior and posterior teeth in the RD, SE, and ZOO groups were significantly lower than those in the control (C) group (P <0.001). There was no significant difference between RD and SE (Anterior P = 0.277; Posterior P = 0.639), whereas ZOO showed significantly lower humidity than both RD (Anterior P = 0.005; Posterior P = 0.003) and SE (Anterior P = 0.002; Posterior P <0.001).

Under low external humidity (L), intraoral humidity levels for both anterior and posterior teeth in the RD, SE, and ZOO groups were significantly lower than those in the control (C) group (P <0.001). Moreover, RD and ZOO demonstrated significantly lower humidity compared to SE (Anterior: RD vs SE P <0.001, ZOO vs SE P <0.001; Posterior: RD vs SE P <0.001, ZOO vs SE P <0.001). No significant differences were observed between RD and ZOO (Anterior P = 0.634; Posterior P = 0.527). The effect sizes of RD and ZOO indicated 1.49 and 1.41, respectively, and the statistical power was 0.99 for both groups.

### Relationship Between External and Intraoral Humidity

**Fig 4:**
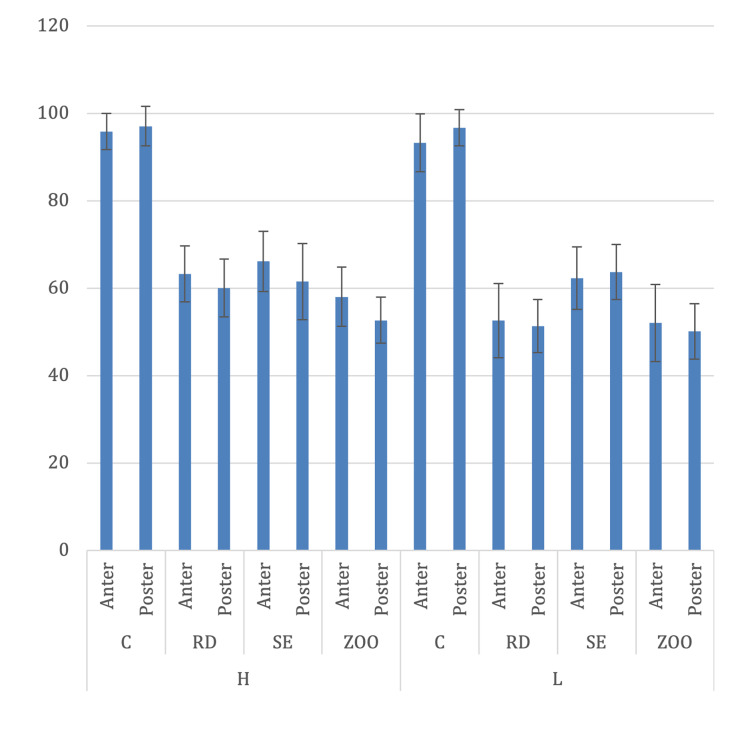
Correlation analysis results between external humidity and intraoral humidity under different moisture control methods. The horizontal axis represents external humidity during measurement (unit: %), while the vertical axis represents intraoral humidity (unit: %).

Figure 4 illustrates the correlation between external and intraoral humidity levels for different moisture control methods.

For all conditions, RD showed strong correlations (Anterior ρ = 0.691, P < 0.001; Posterior ρ = 0.766, P < 0.001). ZOO showed a weaker but significant correlation in anterior teeth (ρ = 0.386, P = 0.014). No significant correlations were observed for SE (Anterior ρ = 0.267; Posterior ρ = 0.746) or control (Anterior ρ = 0.105; Posterior P = 0.097).

For high external humidity (H), RD remained significant (Anterior ρ = 0.716, P = 0.003; Posterior ρ = 0.655, P = 0.007). No significant correlations were observed for the other groups (all P >0.1).

For low external humidity (L), a significant correlation was observed only in posterior teeth in the RD group (ρ = 0.670, P <0.001). No significant correlations were found for the other groups (all P >0.05).

To provide a clearer interpretation, the correlation strength was classified: RD demonstrated a strong correlation, ZOO a weak to moderate correlation, while SE and control showed no correlation.

## DISCUSSION

Previous studies have reported that increased relative humidity impairs bonding performance, although the magnitude of the effect varied depending on the adhesive system and experimental design.^1,2,3,16,18^ Miyazaki et al^16^ and Plasmans et al^18^ showed decreased bond strength above 80% relative humidity, while Bavbek et al^2^ and Besnault et al^3^ reported system-dependent variability. Dabbagh et al^5^ and Saraiva et al^20^ further indicated that adhesives containing HEMA are particularly susceptible. Collectively, these reports emphasize the importance of moisture control but have been largely limited to laboratory settings.

In this study, all moisture control methods significantly reduced intraoral humidity compared with the control. Notably, both RD and ZOO achieved lower humidity levels than SE under low external humidity, whereas under high external humidity, differences among isolation methods diminished. These findings highlight that the clinical effectiveness of isolation depends not only on the method itself but also on the surrounding environment. The observation that intraoral humidity remained elevated under high ambient humidity, even with RD, underscores the importance of considering seasonal or environmental factors, such as rainy periods or air conditioning, in daily practice.

A further strength of this study was the intrapatient comparison of anterior and posterior sites under identical conditions. Contrary to some previous reports,^20^ no difference in humidity was observed between anterior and posterior teeth, likely due to standardized measurement procedures by a single operator. This suggests that careful control of measurement protocols can reduce variability and enhance reproducibility in clinical humidity studies.

The present study design was chosen to simulate realistic clinical conditions where practitioners must select between different isolation strategies depending on patient factors and procedural requirements. Unlike previous reports that primarily assessed humidity changes under limited conditions, this study directly compared multiple isolation methods in both anterior and posterior teeth within the same clinical setting. By incorporating both intraoral and extraoral humidity factors, the findings provide unique evidence on the clinical relevance of moisture control. To our knowledge, this is the first clinical study to quantify intraoral humidity across different isolation methods while simultaneously evaluating external humidity, thereby addressing an important gap in adhesive dentistry research.

Therefore, the hypothesis of this study was partially accepted. It is important to consider patient comfort and procedural efficiency when selecting a moisture control method. It is recommended to use RD and ZOO selectively according to the specific clinical needs and environmental conditions during restoration placement or prosthesis cementation.

### Clinical Significance

Maintaining a low intraoral humidity is essential for successful adhesive restorations. Both RD and ZOO can be selected based on clinical needs and environmental conditions.

## CONCLUSION

All tested moisture control methods were effective in reducing intraoral humidity, though to different extents. Both rubber dam isolation and vacuum-assisted isolation (ZOO) showed superior performance compared with the saliva ejector. Importantly, high external humidity significantly increased intraoral humidity, indicating that the choice of isolation method alone may not be sufficient to fully control intraoral conditions. The novelty of this study lies in the direct comparison of multiple isolation strategies under clinical conditions while accounting for environmental factors. Clinicians should therefore consider not only the isolation technique but also the external environment when selecting strategies to minimize intraoral humidity.

## REFERENCES
